# Nomophobia and Its Relationship with Social Anxiety and Procrastination in Nursing Students: An Observational Study

**DOI:** 10.3390/nursrep13040140

**Published:** 2023-12-05

**Authors:** Irene Tárrega-Piquer, María Jesús Valero-Chillerón, Víctor Manuel González-Chordá, Irene Llagostera-Reverter, Águeda Cervera-Gasch, Laura Andreu-Pejo, Víctor Pérez-Cantó, Víctor Ortíz-Mallasén, Guillem Blasco-Palau, Desirée Mena-Tudela

**Affiliations:** 1 Nursing Department, Universitat Jaume I, Avda Sos Baynat s/n, 12071 Castelló de la Plana, Spain; al387383@uji.es (I.T.-P.); llagoste@uji.es (I.L.-R.); cerveraa@uji.es (Á.C.-G.); pejo@uji.es (L.A.-P.); ortizv@uji.es (V.O.-M.); al225789@uji.es (G.B.-P.); dmena@uji.es (D.M.-T.); 2 Nursing Department, University of Alicante, Carretera San Vicente del Raspeig s/n, 03080 Alicante, Spain; victor.pc@ua.es

**Keywords:** nursing students, Nomophobia, social anxiety, procrastination

## Abstract

Nomophobia is a phenomenon that describes the fear of not having one’s mobile phone accessible. This study aimed to evaluate the presence of nomophobia among nursing students as well as its relationship with procrastination and social anxiety. Methods: An observational, descriptive, cross-sectional study was conducted in a sample of 308 nursing students. Data were collected using the Nomophobia Questionnaire, Academic Procrastination Scale-Short Form, and Social Anxiety Questionnaire for Adults. Additionally, sociodemographic variables related to academic performance and smartphone use were collected. We performed a descriptive, bivariate, and multivariate analysis of the Nomophobia Questionnaire score. Results: 19.5% (*n* = 60) of the students presented with or were at high risk of nomophobic behaviour. Moreover, nomophobic behaviour was positively correlated with high levels of social anxiety (*p* < 0.001), longer daily smartphone usage time (*p* < 0.001), and a high frequency of smartphone checking in class (*p* < 0.001). The predictive variables for nomophobic behaviour included age, variables related to smartphone use, social anxiety levels, work, procrastination tendency, sex, and self-reported average grade. Conclusion: One out of five students in the sample studied presented with or were at high risk of nomophobic behaviour. Additionally, nomophobic behaviour was associated with social anxiety and variables related to smartphone use. This study was not registered.

## 1. Introduction

Worldwide, especially in developed countries, there have been recent major digital transformations at the societal level, with the development of Information and Communication Technologies (ICTs) being a major driving factor [1].

Mobile phones and the Internet were introduced at the user level in 1994, followed by a dramatic increase in their use. In 2019, for the first time, the number of mobile phone subscriptions exceeded the number of people. In 2022, there were 8.6 billion mobile subscribers worldwide [2]. Moreover, people aged 15–24 years use the Internet 1.24 times more than the rest of the population [3].

The wide range of possibilities offered by smartphones with Internet access and the worldwide widespread use of these technologies have altered social interactions, work/study environments, and other aspects of daily living, including shopping and banking. However, misuse or abuse of ICTs may have adverse consequences [4], including mobile phone addiction, which is defined as “compulsive mobile phone usage” [5], or nomophobia, which is defined as “the fear of being unable to use or being unreachable via one’s smartphone” [6]. 

Nomophobia, which is derived from “no mobile phone phobia”, is a relatively recent concept [7]. Accordingly, it remains in the early research stages [8]. One of the most cited consequences of nomophobia in the literature is anxiety about not having a phone nearby [9,10]. This anxiety can cause continuous distractions [11] and have a negative impact on academic performance [12] due to procrastination derived from inappropriate use of the smartphone, causing dysfunctional behaviours [13,14].

Studies have indicated that nomophobia is more prevalent among women [15] and young people, especially those aged <24 years [7,8,16], which is consistent with the profile of nursing students [17].

Moderate-to-high levels of nomophobia have been reported in nursing students [16,18,19,20]. Additionally, mobile phone abuse and nomophobia can negatively affect the academic and learning environments of nursing students [21]. The intensive use of smartphones has been related to a decrease in concentration, which leads to an increase in academic procrastination [22], thus increasing distractions [18] and leading to poor academic performance [8,23].

The presence of nomophobia in nursing students acquires special relevance, not only because of the consequences derived from this phenomenon that may have repercussions on the care provided during clinical practice but also because of its proximity to their practice as nursing professionals. Among these consequences, previous studies have observed that the presence of nomophobia leads to poorer communication with patients [24] and with other healthcare professionals [25], an increased risk of dysfunctional attitudes [26], as well as increased distractions [18,27,28], which may compromise patient safety [22]. Accordingly, it is important to address the problem of nomophobia in the academic sphere. Therefore, this study aimed to evaluate the presence of nomophobia in nursing undergraduate students at the Universitat Jaume I, as well as to explore related factors.

## 2. Materials and Methods

### 2.1. Design and Sample

This descriptive and cross-sectional observational study was conducted between January and June 2022. We included nursing students at Universitat Jaume I (Castellón de la Plana; Spain).

The study population comprised 480 nursing students (120 students per academic course). The selection criteria were to have an electronic device from which to fill in the data collection booklet and to participate voluntarily and anonymously. Non-probabilistic convenience sampling was conducted by taking advantage of the scheduled classes of the degree program.

### 2.2. Variables and Instruments

We collected the following variables: sociodemographic variables (age, sex, work activity, and self-reported average grade), variables related to smartphone use (daily smartphone usage time and frequency of smartphone checking in class), and questionnaire-related variables (procrastination tendency, nomophobia, and social anxiety).

Procrastination tendency was measured using the Academic Procrastination Scale-Short Form (APS-SF), which has been adapted for nursing students and validated in Spanish [29]. This instrument comprises five items measured on a 5-point Likert scale (1 = totally agree, 5 = totally disagree). The APS-SF showed internal consistency within the population sample (α = 0.842). Given the lack of cut-off values for the questionnaire scores, cluster analysis was performed to determine the number of groups in the sample. A cluster analysis of the APS-SF scores was performed using Ward’s method of grouping and using the squared Euclidean distance as a measure. Once the clustering was performed and the resulting categorised variable scores were saved, differences between groups were confirmed by ANOVA test. APS-SF scores of 5–8, 9–16, and 17–25 points correspond to a low, moderate, and high procrastination tendency, respectively.

Nomophobia was assessed using the Nomophobia Questionnaire (NMP-Q). Specifically, we used the Spanish version adapted for nursing students and validated by Gutiérrez-Puertas et al. [12]. This questionnaire comprises 20 items measured on a 7-point Likert scale (1 = totally agree, 7 = totally disagree). The items were grouped into four dimensions: (1) fear of inability to have immediate access to information; (2) fear of giving up the convenience provided by mobile devices; (3) emotions produced by being unable to remain online; and (4) fear or nervousness for being unable to communicate with other people. The NMP-Q showed global internal consistency in the studied sample (α = 0.945); moreover, the internal consistency of its dimensions was as follows: (1) α = 0.839; (2) α = 0.799; (3) α = 0.937; (4) α = 0.818. This questionnaire has three cut-off points related to the 15th, 80th, and 95th percentiles [30], which yielded four categories (no nomophobia, low risk of nomophobic behaviour, moderate risk of nomophobic behaviour, and nomophobic behaviour). 

Finally, social anxiety was measured using the Social Anxiety Questionnaire for Adults (CASO-A30), which has been validated for university students and translated into Spanish [31]. It comprises 30 items measured with a 5-point Likert scale (1 = no discomfort, 5 = a lot of discomfort) and encompasses five dimensions: (1) interaction with the opposite sex; (2) embarrassment or ridicule; (3) interaction with strangers; (4) public speaking/interaction with people in authority; (5) assertive expression of annoyance, displeasure, or anger. The internal consistency for our sample was α = 0.928; additionally, the internal consistency of its dimensions was as follows: (1) α = 0.835; (2) α = 0.680; (3) α = 0.855; (4) α = 0.874; (5) α = 0.774. Given the lack of cut-off values for the questionnaire scores, cluster analysis was performed to determine the number of groups in the sample. The cluster analysis procedure was the same as described for the APS-SF instrument. For the study sample, scores of 44–75, 76–101, and 102–145 points indicate low, moderate, and high anxiety levels, respectively.

### 2.3. Data Collection

Data were collected through online forms in February 2022, after the first semester exams, which allowed the collection of the average academic grades while the students could still remember them. The study was presented to students in scheduled undergraduate classes, where students were informed of the study’s purpose as well as its voluntary and anonymous nature.

### 2.4. Statical Analysis

Quantitative variables are presented as the mean and standard deviation, while qualitative variables are presented as absolute and relative frequencies. For bivariate analysis, the applicability conditions of the parametric tests were initially checked using the Kolmogorov–Smirnov normality test and the Levene test to study the homoscedasticity. Differences were tested using the Chi-square test for qualitative variables and the Mann–Whitney U test, ANOVA test, or Kruskal–Wallis test, as appropriate, depending on the nature of the variables.

Multivariate ordinal regression analysis was performed to explore the effect of variables on nomophobic behaviour. Due to the absence of previous multivariate regression models in this field of study, this model included variables significantly associated with the outcome variable in bivariate analysis. Next, we performed a bivariate analysis to examine the association between the variables included in the model and the remaining variables. Finally, we designed a location model to study the main effects of all variables and significant bivariate interactions among the included variables in the model. The negative log–log link function was used since the univariate analysis indicated that the lower categories were the most probable. The goodness-of-fit indicators and Nagelkerke’s R-squared values were used to determine the quality of the resulting model.

Statistical analyses were performed using the Statistical Package for Social Sciences (SPSS) version 25. Statistical significance was set at *p* < 0.05.

## 3. Results

### 3.1. Description of the Sample

The mean age of the overall population was 21.63 (±5.249) years. Moreover, 88.6% (*n* = 273) of the students were female, and 15.6% (*n* = 48) were employed. Notably, 57.5% (*n* = 177) of the students owned their first smartphone at the age of 12 or 13 years. Additionally, 41.6% (*n* = 128) of the students reported a daily smartphone usage time of 3–5 h, while 27.9% (*n* = 86) reported that they checked their smartphones eight or more times during class. Finally, 78.6% (*n* = 242) of the students reported having notable academic grades (Table 1).

We found that 22.1% (*n* = 68) of the students showed a high tendency to procrastinate. Based on the overall NMP-Q score, 4.9% (*n* = 15) presented nomophobia, and 14.6% (*n* = 45) were at high risk of nomophobic behaviour, which is consistent with the results obtained in the analysis by dimensions. Regarding social anxiety, 39% (*n* = 120) and 16.6% (*n* = 51) of the students presented a high and low risk of developing anxious behaviour, respectively (Table 2).

### 3.2. Bivariate Analysis Results

As shown in Table 3, nomophobic behaviour was positively associated with the daily smartphone usage time and frequency of smartphone checking in class (both *p* < 0.001). In fact, the number of students with nomophobia or at high risk of nomophobic behaviour increases if the number of hours of daily smartphone use increases. Similarly, the number of students with nomophobia or at high risk of nomophobic behaviour also increases if students consult their smartphones more often during lessons. Additionally, the incidence of nomophobic behaviour or high risk of nomophobic behaviour was relatively higher in students aged 21 or 22 years. Nomophobic behaviour showed a significant positive correlation with social anxiety levels (CASO-A30 scores; *p* < 0.001); however, it was not correlated with the tendency to procrastinate (*p* = 0.073). 

Although no differences were observed between the level of procrastination and nomophobic behaviour, it was possible to observe that those students with a higher level of procrastination used their smartphones a greater number of hours daily (*p* < 0.001), as well as a greater number of times during classes (*p* = 0.016). Similarly, social anxiety was associated with the daily smartphone usage time and frequency of smartphone checking in class (both *p* < 0.001) (Table 4).

### 3.3. Multivariate Analysis Results

Table 5 presents the design of the ordinal logistic regression model and shows the variables included as the main effects and the interactions indicated by the bivariate analysis. The global fit test confirmed improvement of the final model compared with the model in which only the intersection was considered (chi-square test: 271.058; *p* < 0.001). The goodness-of-fit was confirmed using Pearson’s test (chi-square:1063.806, *p* < 0.001) and chi-squared test (chi-square: 343.880, *p* = 1). The non-significant deviation indicated no significant difference between the predicted and observed values. Nagelkerke’s pseudo-R-square value was 0.677, which indicated that the included variables accounted for 67.7% of the variance.

Despite the good results of the model, only some categories of the “self-reported average academic grade” showed a significant negative correlation with nomophobia severity (*p* < 0.001). Consistent with the bivariate analysis, multivariate analysis indicated no significant relationship between the tendency of procrastination and nomophobia (*p* = 0.114), despite the positive correlation with daily smartphone usage time and frequency of smartphone checking in class (both *p* < 0.001). Additionally, the level of social anxiety was not positively correlated with the number of nomophobic behaviour categories (*p* = 0.906).

## 4. Discussion

Nomophobia is a common phenomenon among young people [32] that negatively affects academic performance or social interactions [8,33]. Therefore, it is important to study the prevalence of nomophobia among students to mitigate these negative consequences.

In our study, most students fell within the low-risk percentile for nomophobic behaviour, which is inconsistent with similar reports from previous studies. For example, Gutiérrez-Puertas et al. [12] and Gutiérrez-Puertas et al. [21] reported high nomophobia levels among nursing students, while Çatiker et al. [34] reported moderate levels of nomophobic behaviour. Notably, Gutiérrez-Puertas et al. [12] and Gutiérrez-Puertas et al. [21] did not apply standardised categorisation as proposed by González-Cabrera et al. [30], which was used in the present study. Instead, they simply relied on the mean score being above the median of the possible range of questionnaire scores. Çatiker et al. [34] based their categorisation of the different levels of nomophobia on an ad hoc predetermined classification methodology. In the validation process of an instrument, it is useful to establish cut-off points that facilitate the comparability of the results of different studies, since it is in the lack of use of the cut-off points of the NMP-Q where the potential difference between the results described lies. It would be interesting to advance the validation process of the instrument in future studies, so that cut-off points could be established using consistent methodological tests such as the area under the curve.

Regarding the relationship between anxious behaviour and nomophobia, Mir and Akhtar [11] observed a positive correlation between anxiety levels and nomophobic behaviour, which is consistent with our findings. Additionally, they found that nomophobia in individuals with certain cognitive and sensory distractions worsened their anxiety levels. This could be attributed to a fear of missing out on things (FOMO) resulting from not having a mobile phone, which causes a feeling of nervousness and leads to anxious behaviour [35].

Consistent with the reports by Rengifo-Acho and Arapa-Turpo [36], we observed no relationship between the tendency to procrastinate and levels of nomophobia. This could be attributed to the fact that procrastination is not only related to mobile phone addiction but also may occur for other reasons, including excessive social activities. Consistent with the results by Estremadoiro-Parada and Schulmeyer [37], most students exhibited a low-to-moderate tendency to procrastinate. Additionally, in line with the reports by Gutiérrez-Puertas et al. [21] and Ortega Sanz and Dominguez Lara [38], intensive smartphone use during the day and specifically during classes reduces students’ attention and increases procrastination, which negatively affects academic performance [39].

Consistent with the findings by Gutiérrez-Puertas et al. [16] and Çatiker et al. [34], the daily smartphone usage time and frequency of smartphone checking in class were related to nomophobia levels. This is further indicated by the fact that none of the students who used their mobile phones for < 1 h presented nomophobic behaviour. 

An integrative literature review conducted by Ramjan et al. [14] showed that one study observed positive correlations between smartphone addiction and anxiety. Nonetheless, there have been inconsistent reports regarding the relationship of smartphone-related variables with anxiety and nomophobia. Future studies are warranted to elucidate the interactions.

Regarding academic performance, the self-reported average academic grade was not related to nomophobia levels. However, Mendoza et al. [39], Rodríguez-García et al. [8], and Gutiérrez-Puertas et al. [21] observed a significant relationship between nomophobia levels and academic performance. 

Furthermore, Rodríguez-García et al. [8] suggested that variables such as sex and age are predictors of nomophobia; specifically, they observed high levels of nomophobia among nursing students, which is inconsistent with our findings. This suggests that sex is not correlated with the level of nomophobia. 

No previous study on nomophobia levels among nursing studies has performed multivariate analysis, which impedes comparisons of our findings to previous ones. Nonetheless, our findings indicated that the development of nomophobic behaviour is a multifactorial phenomenon. Future well-designed studies are warranted to establish causal relationships. For example, large-scale longitudinal studies are warranted to explore the variables involved and the interactions between them in order to inform interventions for mitigating the development of nomophobic behaviour in nursing students.

It seems interesting to incorporate, from the academic sphere, awareness-raising sessions on the misuse and abuse of technology. Monitoring the use of technology in general and of smartphones, in particular, could be a good starting point from which to become aware of actual individual use. In addition, it would be appropriate to introduce tools that make it easier to manage tasks properly, allowing, for example, temporary distraction blockers to be set up in order to reduce the risk of procrastination through technology. Similarly, in a world that is increasingly connected through technology, appropriate policies on the use of technology in both academic and clinical settings should be established, and access to self-assessments of emotional and mental well-being in relation to technology use should be made available to assess the prevalence of nomophobia.

### Limitations

First, this single-centre study was conducted using a non-randomised sample, which limits the generalisability of our findings. Second, we did not perform a longitudinal analysis of the students throughout the program. Therefore, it was not possible to confirm whether the presence of nomophobia negatively affects the learning environment of nursing students, so it would be interesting to explore this hypothesis in future, more methodologically rigorous studies. Similarly, future studies should consider other variables related to nomophobia levels and smartphone use, including sleep quality, self-esteem, loneliness, and communication skills. Despite these limitations, our findings could inform interventions for nomophobic behaviour among young people, which can have negative effects that extend to the professional stage, and thus affect patient care in healthcare practice.

## 5. Conclusions

Our findings indicated that one in five students nursing undergraduate students in Universitat Jaume I presented with or were at high risk of nomophobic behaviour. Additionally, we identified the following as potential risk factors for nomophobic behaviour: high levels of social anxiety, daily smartphone usage time > 1 h, frequency of smartphone checking in class > 8 times, age of 21 or 22 years, and age at onset of smartphone use of 11–13 years.

## Figures and Tables

**Table 1:** Sociodemographic variables and variables related to smartphone use.

Variable/Category	*n* ^1^	% ^2^
Sex		
Male	35	11.4
Female	273	88.6
Active in employment		
No	260	84.4
Yes	48	15.6
Self-reported average academic record		
Sufficient	49	15.9
Notable	242	78.6
Excellent	17	5.5
Age at which the first smartphone was owned		
<10	11	3.6
10	12	3.9
11	35	11.4
12	116	37.7
13	61	19.8
14	31	10.1
15	18	5.8
16	12	3.9
17	3	1
≥18	9	2.9
Daily cell phone usage time		
<1	15	4.9
1–3	113	36.7
3–5	128	41.6
>5	52	16.9
Times the smartphone is checked during class		
0–3	107	34.7
4–7	115	37.3
≥8	86	27.9

^1^ Absolute frequencies; ^2^ Relative frequencies.

**Table 2:** Descriptive analysis of questionnaires.

Questionnaire/Category	*n* ^1^	% ^2^
Academic Procrastination Scale-Short Form (APS-SF)		
Low procrastination tendency	70	22.7
Middle procrastination tendency	170	55.2
High procrastination tendency	68	22.1
Nomophobia Questionnaire (NMP-Q)		
No nomophobic behaviour	48	15.6
Low risk of nomophobic behaviour	200	64.9
High risk of nomophobic behaviour	45	14.6
Nomophobic behaviour	15	4.9
Social Anxiety Questionnaire for Adults (CASO-A30)		
Low risk of anxious behaviour	51	16.6
Middle risk of anxious behaviour	137	44.5
High risk of anxious behaviour	120	39

^1^ Absolute frequencies; ^2^ Relative frequencies.

**Table 3:** Relationships between nomophobic behaviour and variables under study.

	No Nomophobic Behaviour		Low Risk of Nomophobic Behaviour		High Risk of Nomophobic Behaviour		With Nomophobic Behaviour		
	*n* ^1^	% ^2^	*n*	%	*n*	%	*n*	%	*p*-Value
Daily cell phone usage hours									<0.001 ^3^
<1	7	46.7	8	53.3	-	-	-	-	
1–3	24	21.2	75	66.4	11	9.7	3	2.7	
3–5	14	10.9	86	67.2	23	18	5	3.9	
>5	3	5.8	31	59.6	11	21.2	7	13.5	
Times the smartphone is consulted in class									<0.001 ^3^
0–3	30	28	69	64.5	5	4.7	3	2.8	
4–6	12	12.4	67	69.1	14	14.4	4	4.1	
7–9	2	5.4	22	59.5	10	27	3	8.1	
≥10	4	6	42	62.7	16	23.9	5	7.5	
Age categorized by percentiles (years old)									0.009 ^3^
18–19	8	8.5	68	72.3	16	17	2	2.1	
20	12	17.4	45	65.2	10	14.5	2	2.9	
21–22	13	13.5	59	61.5	14	14.6	10	10.4	
≥23	15	30.6	28	57.1	5	10.2	1	2	
Age at which the first smartphone was owned (years old)									<0.001 ^3^
<10	-	-	4	36.4	6	54.5	1	9.1	
10	2	16.7	8	66.7	2	16.7	-	-	
11	3	8.6	19	54.3	12	34.3	1	2.9	
12	22	19	78	67.2	10	8.6	6	5.2	
13	7	11.5	42	68.9	9	14.8	3	4.9	
14	4	12.9	24	77.4	3	9.7	-	-	
15	1	5.6	13	72.2	1	5.6	3	16.7	
16	5	41.7	4	33.3	2	16.7	1	8.3	
17	-	-	3	100	-	-	-	-	
≥18	4	44.4	5	55.6	-	-	-	-	
	**m ^4^**	**sd ^5^**	**m**	**sd**	**m**	**sd**	**m**	**sd**	***p*-Value**
Social anxiety	82.5	22.4	96.09	20.42	103.67	17.29	114.07	17.86	<0.001 ^6^
Procrastination	11.21	4.83	12.99	4.66	13.20	4.52	12.13	5.01	0.073 ^6^

^1^ Absolute frequencies; ^2^ Relative frequencies; ^3^ Chi-square test; ^4^ Mean; ^5^ Standard deviation; ^6^ Kruskal-Wallis.

**Table 4:** Relationship of smartphone-related variables with procrastination tendency and social anxiety.

	Social Anxiety			Procrastination		
	m ^1^	sd ^2^	*p*-Value ^3^	m	sd	*p*-Value ^3^
Daily cell phone usage hours			<0.001			<0.001
<1	79.60	25.63		8.93	4.92	
1–3	91.27	21.12		12.15	4.94	
3–5	98.84	21.07		13.35	4.44	
>5	103.77	16.69		13.38	4.21	
Times the smartphone is consulted in class			<0.001			<0.016
0–3	91.63	21.54		11.54	4.78	
4–6	100.95	20.85		12.39	4.50	
7–9	93.19	22.41		14.12	4.37	
≥10	97.16	20.10		14.22	4.56	

^1^ Mean; ^2^ Standard deviation; ^3^ Kruskal-Wallis.

**Table 5:** Ordinal logistic regression model for nomophobic behaviour.

Location Model		Link Function: Negative Log-Log	
**Main Effects**			
Age categorised by percentiles Age at which the first smartphone was owned Times the smartphone is consulted in class Daily cell phone usage hours Social anxiety Active in employment Procrastination Sex Self-reported average academic record			
**Interactions included in the model from the bivariate analysis**			***p-*Value**
Social anxiety × Active in employment			<0.001 ^1^
Social anxiety × Daily cell phone usage hours			<0.001 ^1^
Times the smartphone is consulted in class × Age categorised by percentiles			<0.001 ^2^
Times the smartphone is consulted in class × Daily cell phone usage hours			<0.001 ^2^
Times the smartphone is consulted in class × Procrastination			<0.001 ^1^
Daily cell phone usage hours × Age categorised by percentiles			<0.001 ^2^
Daily cell phone usage hours × Age with the first smartphone			<0.001 ^2^
Daily cell phone usage hours × Procrastination			0.002 ^1^
Social anxiety × Sex			0.003 ^3^
Daily cell phone usage hours × Self-reported average academic record			0.004 ^2^
Social anxiety × Times the smartphone is consulted in class			0.016 ^1^
Social anxiety × Age categorised by percentiles			0.017 ^1^
Daily cell phone usage hours × Active in employment			0.019 ^2^
Times the smartphone is consulted in class × Age with the first smartphone			0.034 ^2^
Procrastination × Self-reported average academic record			0.044 ^4^
**Results**	Logarithm of the likelihood: 343,880 Chi-square test: 271,058; Pearson Chi: 1063,806 Deviation Chi: 343,880 1 Nagelkerke R-square: 0.677		<0.001

^1^ Kruskal–Wallis; ^2^ Chi-square test; ^3^ U de Mann–Whitney; ^4^ ANOVA.

## Data Availability

Data are available upon reasonable request. All necessary data are supplied and available in the manuscript; however, the corresponding author will provide the dataset upon request. All data relevant to the study are included in the article.

## Public Involvement Statement

No public involvement in any aspect of this research.
